# Receptor-Mediated Delivery of Astaxanthin-Loaded Nanoparticles to Neurons: An Enhanced Potential for Subarachnoid Hemorrhage Treatment

**DOI:** 10.3389/fnins.2019.00989

**Published:** 2019-09-18

**Authors:** Zong-qi You, Qi Wu, Xiao-ming Zhou, Xiang-sheng Zhang, Bin Yuan, Li-li Wen, Wei-dong Xu, Sheng Cui, Xiang-long Tang, Xin Zhang

**Affiliations:** ^1^Department of Neurosurgery, School of Medicine, Jinling Hospital, Jiangsu University, Zhenjiang, China; ^2^Department of Neurosurgery, School of Medicine, Jinling Hospital, Nanjing University, Nanjing, China; ^3^Department of Neurosurgery, School of Medicine, Jinling Hospital, Second Military Medical University, Shanghai, China; ^4^Department of Neurosurgery, School of Medicine, Jinling Hospital, Nanjing Medical University, Nanjing, China; ^5^Department of Neurosurgery, School of Medicine, Jinling Hospital, Southern Medical University (Guangzhou), Nanjing, China; ^6^College of Material Sciences and Engineering, Nanjing Tech University, Nanjing, China

**Keywords:** astaxanthin, transferrin, nanoparticle, targeted delivery, neuroprotection

## Abstract

Astaxanthin (ATX) is a carotenoid that exerts strong anti-oxidant and anti-inflammatory property deriving from its highly unsaturated molecular structures. However, the low stability and solubility of ATX results in poor bioavailability, which markedly hampers its application as therapeutic agent in clinic advancement. This study investigated a promising way of transferrin conjugated to poly (ethylene glycol) (PEG)-encapsulated ATX nanoparticles (ATX-NPs) on targeted delivery and evaluated the possible mechanism underlying neuroprotection capability. As a result, the ATX integrated into nanocarrier presented both well water-dispersible and biocompatible, primely conquering its limitations. More than that, the transferrin-containing ATX-NPs exhibited enhanced cellular uptake efficiency than that of ATX-NPs without transferrin conjugated in primary cortical neurons. Additionally, compared to free ATX, transferrin-containing ATX-NPs with lower ATX concentration showed powerful neuroprotective effects on OxyHb-induced neuronal damage. Taken together, the improved bioavailability and enhanced neuroprotective effects enabled ATX-NPs as favorable candidates for targeted delivery and absorption of ATX. We believe that these *in vitro* findings will provide insights for advancement of subarachnoid hemorrhage therapy.

## Introduction

Subarachnoid hemorrhage (SAH) is one of the most severe medical emergencies of stroke suffered from lysed blood in the subarachnoid space surrounding the brain (Zacharia et al., 2010). Early brain injury (EBI), which typically develops within 72 h of SAH, is considered as the major cause of disability and death in humans (Sehba et al., 2012). Recently, more and more evidences suggest that neuronal apoptosis, which causes cerebral cortex damage and aggravates neurobehavioral impairments, has become an important factor in pathophysiologic events of EBI and may account for the serious impacts on short- as well as long-term clinical prognosis (Matz et al., 2000; Sabri et al., 2008). Therefore, embracing anti-apoptosis should be potential therapeutic approach for preventing the aggravation of EBI.

**FIGURE 1:**
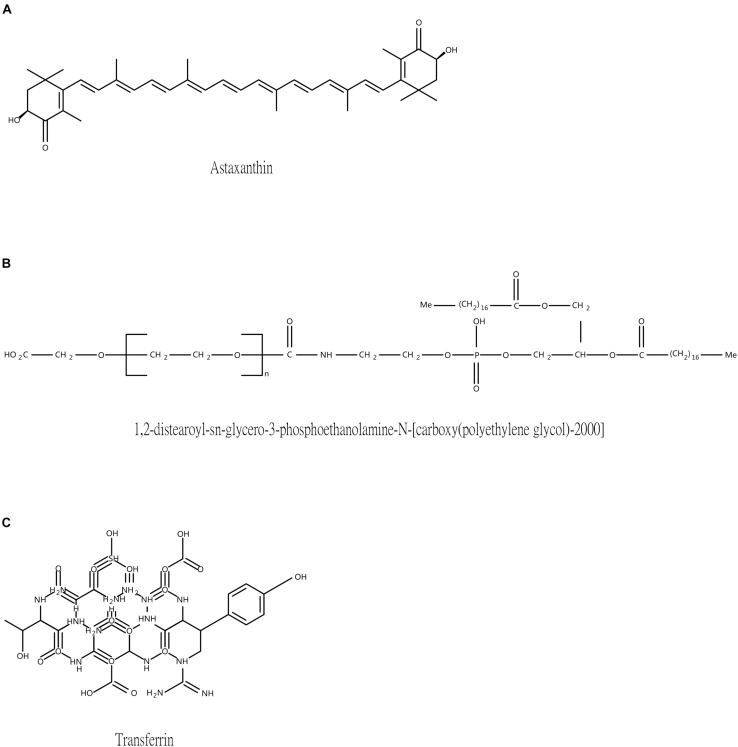
The structure of three main ingredients. **(A)** Astaxanthin **(B)** DSPE-PEG-COOH **(C)** Transferrin.

Astaxanthin (ATX), composed of conjugate double bond and hydroxyl group (Figure 1), is a carotenoid pigment naturally distributed in aquatic animals and various plants (Fassett and Coombes, 2009). According to previous studies (Wu et al., 2014; Zhang et al., 2014a, b, c; Zhang et al., 2015), our laboratory group has proved that ATX exerts formidable anti-oxidant, anti-inflammatory, and anti-apoptotic properties against EBI and neurotoxin-induced neurotoxicity after experimental SAH both *in vivo* and *in vitro*. The hydrophobic property, however, restricted its effects in terms of oral intake (Zhang et al., 2014c), and the granular medicine neither dispersed well intravenously nor enhanced the efficiency for cellular uptake (Kulkarni and Feng, 2013). Despite several approaches have been attempted to improve the solubility and stability of ATX such as liposomes, nanoemulsion and colloidal particles (Peng et al., 2010; Redzuan et al., 2011; Anarjan et al., 2012), effective nanoencapsulation of ATX has not yet been evaluated for cerebral treatment.

In the previous work, we synthesized albumin-loaded Fe_3_O_4_ nanoparticles (NPs) encapsulated with paclitaxel as drug delivery vehicles toward C6 cell lines *in vitro* effectively (Tang et al., 2016), providing broad therapeutic prospects for glioma treatment. As for EBI therapy after SAH, here, targeting technology presents a challenge for effective entry of therapeutic drugs. To our knowledge, transferrin receptor is known as specific transporter in blood-brain barrier (BBB) that allows transferrin to attach to and cross the intracranial barrier (Moos and Morgan, 2000). Interestingly, we also found that it was mainly located in neuron membranes compared to the other cells in the central nervous system (CNS) (Moos, 1996). To date, increasing evidences have supported the utility of transferrin receptor in the arena of nanomedicine delivery to improve oral bioavailability, to enhance transfer across the BBB, and to reinforce delivery of therapeutics to selective cell types for cerebral diseases (Jiang et al., 2012; Dixit et al., 2015), whereas, in reference to precise treatment for neuronal injury, little has been investigated.

**FIGURE 2:**
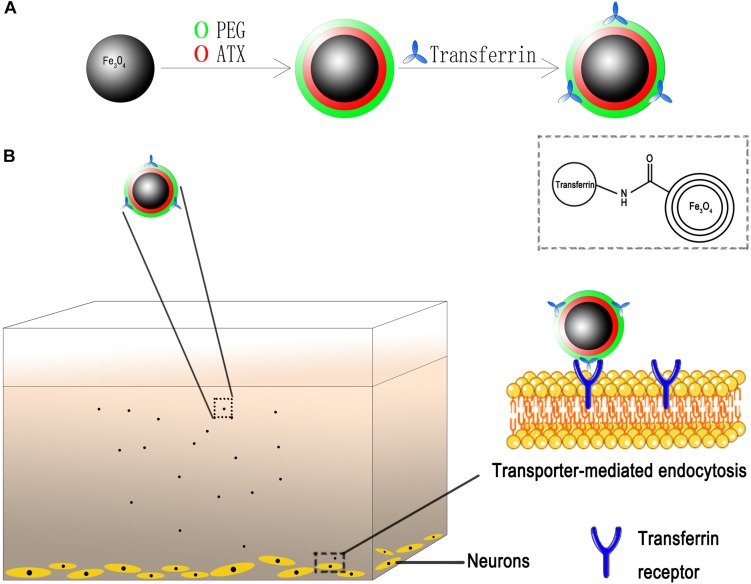
Schematic of ATX-NPs delivery system. **(A)** Design of transferrin conjugated to PEG-encapsulated astaxanthin nanoparticles (ATX-NPs). **(B)** An *in vitro* model with the neurons grown on the bottom of the well. ATX-NPs with covalently attached transferrin were recognized and internalized by transferrin receptors of neurons. The figure was a simplified representation of complex mechanisms and their interaction. More details for stages of internalization are presented in Figure 8.

Herein, on basis of our technology, we developed desired NPs loaded with ATX and encapsulated by PEG as the gatekeeper. Then, the transferrin molecular was covalently attached to the PEG layer via carbodiimide reaction. The schematic of the design is shown in Figure 2. We also for the first time elucidated the molecular mechanisms underlying the transferrin-receptor mediated endocytosis by ATX-NPs. To appraise diverse cellular uptake properties, NPs modified with transferrin were compared with unmodified ones using cortical neuron culture model. Further, due to the neurotoxic effect from heme moiety, the release of oxyhemoglobin (OxyHb) in subarachnoid space after SAH leads to the cell necrosis in the cortex to a great extent (Pluta et al., 1998; Lara et al., 2009). As a major component of blood, OxyHb provides reactive oxygen species (ROS) and heme and has been widely used as an inducer of SAH model *in vitro* in previous studies (Ishiguro et al., 2006; Sun et al., 2014; Zhang et al., 2018). In this model, thus, the study was conducted to explore the efficacy of our transferrin-containing ATX-NPs for neuronal uptake and neuroprotection potentials for SAH treatment.

## Materials and Methods

### Materials and Reagents

All reagents applied in this study were of analytic grade and did not need to be further purified.

Ferric chloride hexahydrate (FeCl_3_⋅6H2O), sodium oleate (C_17_H_33_COONa, 95%), n-hexane, ethanol, oleic acid (C_17_H_33_COOH), and 1-octadecene (C_18_H_36_, 90%) were purchased from Wanqing Chemical Corporation (Jiangsu, China). N-hydroxysuccinimide (NHS) was procured from damas-beta (Shanghai, China). 1-Ethyl-3-(3-(dimethylaminopropyl) carbodiimide (EDC), MES sodium salt, ATX (≥97%, HPLC), transferrin (recombinant), oxyhemoglobin (OxyHb), 4,6-Diamidino-2-phenylindole (DAPI), rabbit anti-β-actin, rabbit anti-Bax antibody, rabbit anti-Bcl-2 antibody and rabbit anti-cleaved caspase-3 antibody were purchased from Sigma-Aldrich (St. Louis, MO, United States). DSPE-PEG2000-COOH was purchased from A.V.T Pharmaceutical corporation (Shanghai, China). Alexa Fluor 488 conjugated anti-MAP2 antibody was purchased from Merck Millipore (Darmstadt, Germany). Alexa Fluor 647 NHS Ester was purchased from Thermo Fisher Scientific (Waltham, MA, United States).

### Animal Preparation

All procedures were approved by the Animal Care and Use Committee of Jiangsu University and were carried out according to the Guide for the Care and Use of Laboratory Animals published by National Institutes of Health. Primary cortical neurons were prepared from the pups of fifteen- to eighteen-day-old gestational C57BL/6 mice, which were purchased from the Animal Center of Jinling Hospital (Nanjing, China).

### Synthesis of Stabilized Fe_3_O_4_ NPs

The Fe_3_O_4_ NPs were synthesized by a thermal decomposition method (Tang et al., 2016). Firstly, sodium oleate and ferric chloride were dissolved with the mole ratio of 3:1 in a mixture containing hexane, distilled water and ethanol. Then the as-obtained complex was heated at 70°C for 4 h followed by washed and dried in vacuum for 24 h. After that, we dissolved oleate acid (3.1 g) and iron oleate complex (20 g) in 1-octadecene with evenly stirring at ambient temperature. Next, the solution was slowly heated to 250°C and maintained for 1 h. Afterward, the mixture continued to be heated to 320°C and maintained the temperature for another 45 min. The reactants were finally cooled to ambient temperature and ethanol (500 ml) was added in company. After centrifugation at 7500 rpm for 10 min, the precipitation of Fe_3_O_4_ nanocrystals was obtained and washed three times using ethanol and hexane mixture solution (v:v, 1:1).

### Synthesis of Stabilized Fe_3_O_4_/ATX NPs

For conjugation, ATX was directly absorbed on the particle surfaces and encapsulated with DSPE-PEG-COOH, mediated mainly by hydrophobic interaction. Briefly, as-prepared oleate coated Fe_3_O_4_ NPs (5 ml) and ATX (100 mg) were dissolved in chloroform (20 ml) and stirred for 30 min at room temperature. Then 100 mg DSPE-PEG-COOH powder was added together with constantly stirring for 30 min at ambient temperature. Next, we transferred the mixture to a 50 ml round-bottom flask and added deionized water (5 ml) together. Eventually, the chloroform in the mixture was rotarily evaporated at 70°C until completely vaporized and the monodispersed water-soluble Fe_3_O_4__/_ATX NPs were prepared.

### Synthesis of Stabilized Fe_3_O_4_/ATX/Transferrin NPs

In the fabrication of Fe_3_O_4_/ATX/Transferrin, transferrin molecules were chemically conjugated to Fe_3_O_4_/ATX. In detail, 10 mg as-synthesized Fe_3_O_4_/ATX NPs were suspended in MES buffer (0.02 M) by ultrafiltration centrifugation at 4500 rpm for 7 min. Then, carboxyl groups onto the NPs were activated by EDC (29 mg) and NHS (25 mg) in MES buffer (20 ml) followed by stirring for 30 min. Next, the mixture was ultrafiltrated for three times and dispersed in borate buffer (0.02 M) with transferrin molecules (0.5 mg). After stirring constantly for 5 h, free transferrin was removed by ultrafiltration with deionized water. As a result, the product was filtered and stored in 4°C.

### Particle Size, Zeta Potential, and Entrapment Efficiency of ATX-NPs

The morphology of Fe_3_O_4_/ATX and Fe_3_O_4_/ATX/Transferrin was evaluated using TEM (Tokyo JEOL, Japan). Dynamic light scattering (DLS) (Malvern ZS90, United Kingdom) was used for measuring the hydrodynamic size distribution. The iron concentration of the resulting NPs was analyzed on a UV-vis spectrophotometer (UV-3600, Shimadzu, Japan) by 1, 10-phenanthroline spectrophotometric method. The excitation and emission wavelength were detected on a fluorescence spectrometer (HJY-FL3-211-TCSPC, France). Bicinchoninic acid (BCA) kit (Beyotime Biotechnology, Shanghai, China) was used to evaluate the coupling content of transferrin on NPs by the microplate reader (BioTek ELx808, United States). The standard concentration and non-encapsulated amounts of ATX were measured at OD430 nm by the same microplate reader, calculating the entrapment efficiency of ATX-NPs.

$$\mathrm{ATX}\mathrm{entrapment}\mathrm{efficiency}\left(\%\right)=\frac{\mathrm{total}\,\mathrm{amount}\,\mathrm{of}\,\mathrm{ATX}-\mathrm{non}-\mathrm{envapsulated}\,\mathrm{ATX}}{\mathrm{total}\,\mathrm{amount}\,\mathrm{of}\,\mathrm{ATX}}\times 100$$

Mean values were reported from three individual experiments.

### Primary Neuron Culture

Primary cortical neurons were cultured from fifteen- to eighteen-day-old gestational C57BL/6 mice as previously described (Sun et al., 2014; Zhang et al., 2017). In detail, cerebral cortex was isolated from brains of fetal mice at first. Then, under the microscope, the meninges and blood vessels were stripped, and residual cortical tissues were digested using 0.25% trypsin (Gibco, CA, United States) for 5 min at 37°C. Next, the supernatant was discarded and the digested tissues were washed with pre-cooling phosphate buffered saline (PBS). Afterward, the suspensions were filtered by 22 μm-filter. After centrifugation at 1500 r/min for 10 min, the cells were seeded on poly-D-lysine-coated 6-well plates at a density of 1 × 10^6^/cm^2^ and 12-well plates at a density of 1 × 10^4^/cm^2^ then suspended in Neurobasal Media containing B27, glutamate, Hepes and penicillin-streptomycin (Gibco, CA, United States). Cultured neurons were maintained in a humidified incubator (5% CO_2_, 37°C). One half of the medium was replaced with fresh one every 2 days. Finally, the primary cortical neurons which have been in culture for about 10 days were used *in vitro* studies.

### Efficiency of Uptake of ATX-NPs by Primary Cultured Neurons

To investigate the targeted delivery of Fe_3_O_4_/ATX/Transferrin, cellular uptake experiments were performed with primary cultured neurons. Fixed doses of Fe_3_O_4_/ATX and Fe_3_O_4_/ATX/Transferrin (12.0 μg/ml, corresponding to 20 μM ATX) were administered to the neurons respectively. The primary cultured cortical neurons were randomly divided into two groups: Fe_3_O_4_/ATX group and Fe_3_O_4_/ATX/Transferrin group. The neurons were collected after 6 h incubation at 37°C.

For Laser Confocal Fluorescence Microscopy (LSCM) analysis, the neurons were firstly fixed with 2% paraformaldehyde. After washed with PBS for three times, they were incubated with 0.1% Triton X-100 and 5% FBS in succession. Then, the sections were incubated with MAP2 for 12 h. After washed with PBST, the sections were stained with DAPI for 10 min at ambient temperature. In this work, Alexa Fluor 647 dye as a marker of nanoparticles is a bright, far-red–fluorescent dye with excitation for the 594 or 633 nm laser lines. MAP2 (microtubule associated protein-2), which appears to be green-fluorescent, is a stringent marker for neurons and displays intracellular specificity. DAPI (4′,6-diamidino-2-phenylindole) is a blue-fluorescent DNA stain upon binding to AT regions of dsDNA. Images were obtained using a laser confocal scanning microscope (Leica TCS SP8, Germany) and the fluorescently stained cells were analyzed by Image J software.

For Biological Transmission Electronic Microscopy (BTEM) analysis, primary cultured neurons were fixed with 2.5% glutaraldehyde overnight at first. Then they were cut into small pieces of 1 mm^3^. After that, the specimens were stained with 1% uranyl acetate overnight in the dark. The next day, the stained samples were further dehydrated by ethanol alcohol with increasing concentrations from 25 to 100%. Finally, the neurons which were completely dehydrated were embedded in resin and cured in the oven (60°C) for 2 days for TEM observation.

### Evaluation of Efficacy of ATX-NPs in Inhibiting OxyHb-Induced Neuronal Apoptosis

To investigate the neuroprotective effects of ATX-NPs, OxyHb incubation in cultured neurons was employed as the *in vitro* model of experimental SAH in the present study. Firstly, primary cortical neurons were pre-treated with native ATX and ATX-NPs (fixed dosage of 6.7 μg/ml, corresponding to about 11 μM ATX), respectively, and then were incubated with OxyHb dissolved in culture medium at final concentration of 10 μM. Next, on account of transferrin contrast, they were randomly divided into four groups: control group, SAH group, SAH + Fe_3_O_4_/ATX group and SAH + Fe_3_O_4_/ATX/Transferrin group. Further, comparison between native ATX and ATX-NPs was examined in a dose-based manner as previously reported (Zhang et al., 2019). The cultured neurons were as such divided forward to five groups: control group, SAH group, SAH + 10 μM ATX group, SAH + 50 μM ATX group and SAH + Fe_3_O_4_/ATX/Transferrin group. Finally, the neurons were collected at 12 h after OxyHb exposure.

For western blot analysis, the samples from primary cultured neurons were lysed in radioimmunoprecipitation assay buffer (RIPA) (Beyotime Biotechnology, Shanghai, China) containing phosphatase and protease inhibitor (Roche, Mannheim, Germany). Protein concentrations were estimated using the BCA kit (Beyotime Biotechnology, Shanghai, China). The whole process of experiments was conducted as previously described (Zhang et al., 2017). In brief, equal concentration samples were separated by 10% SDS-PAGE and electro-transferred onto polyvinylidene difluoride (PVDF) membrane (Bio-Rad Lab, Hercules, CA, United States). The membrane was blocked with 5% skim milk for 2 h at ambient temperature and incubated overnight at 4°C with primary antibodies against β-actin (diluted 1:5000), Bcl-2 (diluted 1:500), Bax (diluted 1:500), and cleaved caspase-3 (diluted 1:1000). Following that, they were incubated with proper horseradish peroxidase-conjugated secondary antibodies (Jackson ImmunoResearch Laboratories, PA, United States). The immunoreactive bands were visualized with enhanced chemiluminescence (ECL) reagent kit (Millipore, Darmstadt, Germany). Band intensities were quantified using the ImageJ software.

For flow cytometry analysis, the experiment was performed as follows: Primary cultured neurons were firstly washed with PBS for three times. Then they were resuspended in fluorescein isothiocyanate (FITC)-conjugated Annexin-V binding buffer. After that, we added 5 μl FITC-conjugated Annexin-V (Keygen Biotech, Jiangsu, China) together avoiding light exposure and the reactants were maintained for 15 min at ambient temperature. At last, 5 μl propidium iodide (PI) was added with incubation for 10 min and the neurons were analyzed by flow cytometer (Becton-Dickinson FACSCalibur, United States).

### Cytotoxicity Analysis

The cytotoxicity assay of ATX-NPs on neurons was studied by measuring lactate dehydrogenase (LDH) activity. Briefly, neurons were separately incubated with Fe_3_O_4_/ATX and Fe_3_O_4_/ATX/Transferrin (increasing concentrations for 6.0, 12.0, and 24 μg/ml) and then operated using LDH kit (Beyotime Biotechnology, Shanghai, China) according to the protocol. At last, the supernatant was collected and the OD value at 490 nm was measured by spectrophotometer.

### Statistical Analysis

All data in this experiment were expressed as the mean ± standard error of mean (SEM). Statistical analysis was performed by SPSS 25.0 software (Inc., Chicago, IL, United States). Differences between two groups were evaluated by Student’s *t* test and one-way analysis of variance (ANOVA) was used for more than two groups. Possibility less than 0.05 (*p* < 0.05) was considered significant.

## Results

### Physicochemical Properties and Morphology of ATX-NPs

**FIGURE 3:**
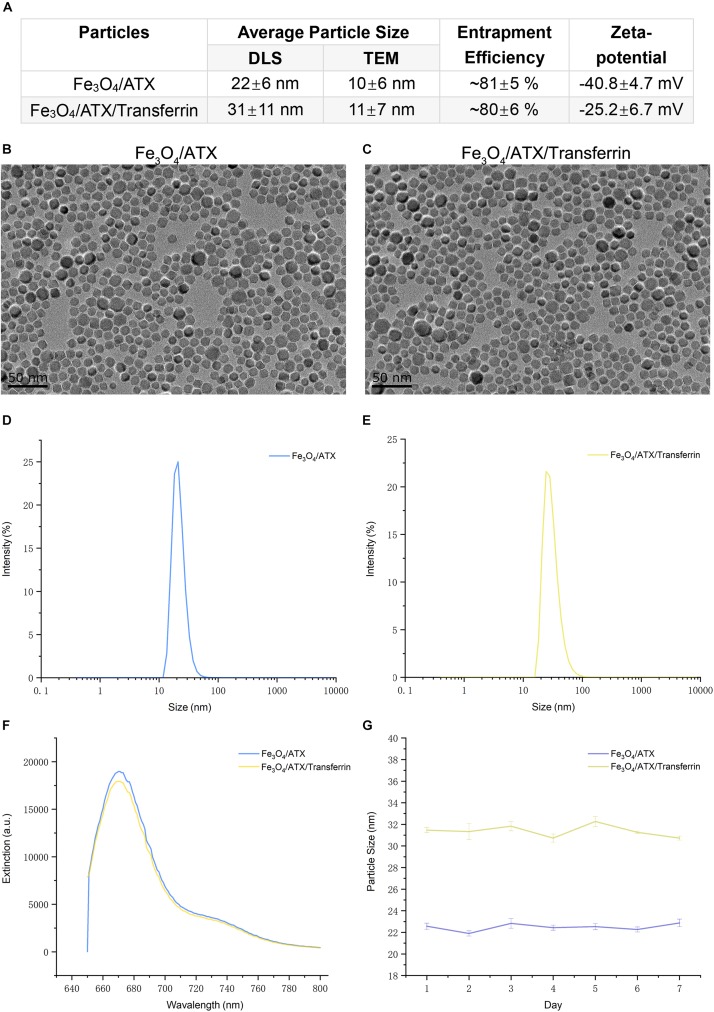
Physicochemical characteristics of ATX-NPs. **(A)** The average particle size, entrapment efficiency, and zeta-potential of ATX-NPs. **(B,C)** Representative TEM pictures of ATX-NPs. **(D,E)** Dynamic laser scattering (DLS) measurement of hydrodynamic size distribution of ATX-NPs. **(F)** Fluorescence emission spectra of ATX-NPs. **(G)** Particle size of ATX-NPs during 7 days storage at 4°C. The values represent the mean ± SEM.

This study brought off a reactive stabilizer for NPs preparation, enabling an effective modification onto ATX-NPs surface. Thus, we made use of PEG for the formulation of ATX-NPs by solvent evaporation method (Figure 2). ATX was insoluble in water; by contrast, ATX-NPs monodispersed well in water. The carboxyl groups of PEG were conjugated to the amino groups of transferrin using carbodiimide reaction, as a result of the covalent linkage. BCA assay revealed that approximately 86 μg transferrin was coupled to NPs (per mg, in accordance with Fe content). After synthesis, ATX-NPs were characterized using both TEM and DLS (Figure 3). The constructed formulations presented particle size of 22 nm (Fe_3_O_4_/ATX), 31 nm (Fe_3_O_4_/ATX/Transferrin) and negative zeta potential of −40.8 mV (Fe_3_O_4_/ATX), −25.2 mV (Fe_3_O_4_/ATX/Transferrin), respectively (Figure 3A). The entrapment efficiency was severally calculated to be about 81 and 80% (Figure 3A).

Figures 3D,E showed a narrow size distribution of Fe_3_O_4_/ATX and Fe_3_O_4_/ATX/Transferrin ranging from 10 to 100 nm. According to the TEM, the ATX-NPs were observed to be near-spherical with homogenous size distribution (Figures 3B,C). The fluorescence spectrum analysis of ATX-NPs (from 650 to 800 nm) with sharp fluorescence peak at 670 nm was conducted toward red spectrum (Figure 3F).

The stability of ATX-NPs is important because of the expiration date of a particular formulation. The optimized ATX-NPs were stored at 4°C away from light and evaluated for particle size for a week. There were negligible alterations in the values of optimized ATX-NPs after storage for a week under the given conditions. Results from DLS showed no differences in the size of a week (Figure 3G). The results indicated that the developed ATX-NPs were physically stable and retained their pharmaceutical properties.

### ATX-NPs Uptake by Primary Cultured Neurons

**FIGURE 4:**
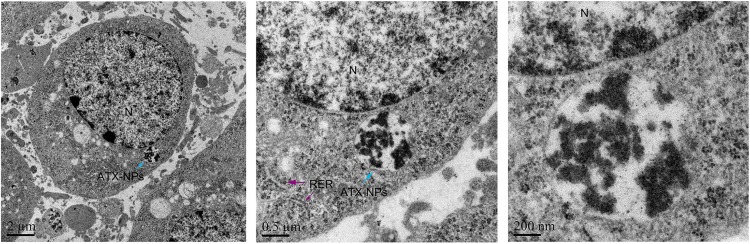
TEM photographs showed uptake of ATX-NPs in the cytoplasm of neurons. Scale bars are 2 μm, 0.5 μm, and 200 nm.

We have demonstrated that transferrin could be effectively conjugated to ATX-NPs by EDC/NHS method. To determine the efficiency of NPs uptake by primary cultured neurons, AF647-labeled ATX-NPs were co-cultured with neurons for 6 h and subsequently analyzed by TEM and LSCM. TEM images provided powerful evidence that ATX-NPs could be primely internalized into neurons and the intracellular endosomes were explicitly shown in Figure 4. In detail, either Fe_3_O_4_/ATX or Fe_3_O_4_/ATX/Transferrin was evidently located inside cytoplasm rather than nucleus. Additionally, photomicrographs with high magnification in Figure 4 indicated the fine structure of ATX-NPs inside neurons.

**FIGURE 5:**
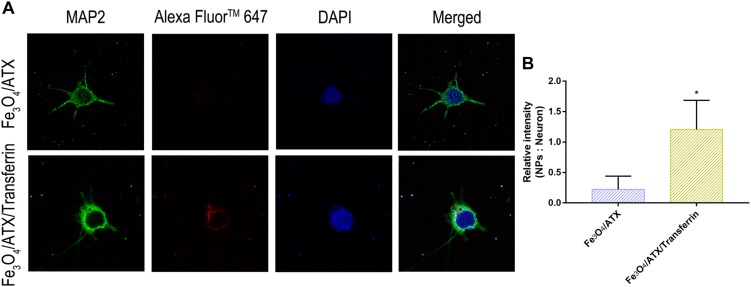
Comparison of neuronal uptake between Fe_3_O_4_/ATX and Fe_3_O_4_/ATX/Transferrin. **(A)** Typical confocal images showed primary cultured neurons in Fe_3_O_4_/ATX group and Fe_3_O_4_/ATX/Transferrin group at 6 h after incubation. **(B)** The intensity in relative fluorescence of Nanoparticles: Neurons. Images were standardized to the same setting. The green, the blue, and the red indicates fluorescence of MAP2, DAPI, and AF647-labeled NPs, respectively. Values are expressed as the means ± SEM. ^∗^*p* < 0.05 vs. Fe_3_O_4_/ATX group; ns *p* > 0.05 vs. Fe_3_O_4_/ATX group.

Further, the red fluorescence observed in neurons (Figure 5A) also demonstrated that ATX-NPs could be efficiently transported into neurons and accumulated at its target site. Compared to ATX-NPs without transferrin conjugated, the neurons appeared to uptake more Fe_3_O_4_/ATX/Transferrin (Figure 5B), qualitatively suggesting that the transferrin conjugation was likely to enhance the transcytosis and internalization of ATX-NPs, although more research to quantify internalization was required.

### ATX-NPs Protect Against OxyHb-Induced Neuronal Apoptosis

**FIGURE 6:**
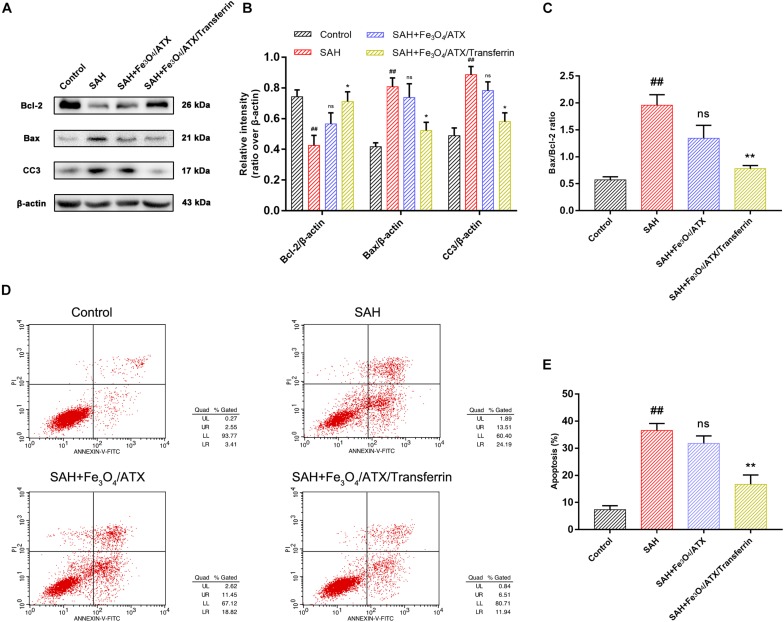
Effects of ATX-NPs on neuronal apoptosis at 12 h post OxyHb exposure. **(A)** Representative Western blots showed the expression of Bcl-2, Bax, and cleaved caspase-3 (CC3) at 12 h after OxyHb exposure in the control group, SAH group, Fe_3_O_4_/ATX-treated group and Fe_3_O_4_/ATX/Transferrin-treated group. **(B,C)** The relative densities of protein bands were analyzed and normalized to β-actin. **(D)** Apoptosis detection results were detected by flow cytometry. **(E)** The ratio of apoptotic cells in each group was measured. Values are expressed as the means ± SEM. ^##^*p* < 0.01 vs. Control group; ns *p* > 0.05 vs. SAH group; ^∗^*p* < 0.05 vs. SAH group; ^∗∗^*p* < 0.01 vs. SAH group.

The balance between Bax and Bcl-2 is fundamental for cell survival and death. With respect to apoptosis, higher expression of Bax may induce permeabilization of mitochondrial membrane, release of cytochrome c and activation of caspase-3 and -9 (Kooijman et al., 2014). In this study, neurons were stimulated with OxyHb to mimic SAH condition. The Western Blot analysis revealed that OxyHb exposure significantly decreased the expression of Bcl-2 and increased the expression of Bax and cleaved caspase-3 at 12 h in neurons when compared with control group (Figures 6A,B). Nonetheless, ATX-NPs administration increased the expression of Bcl-2 and decreased the expression of Bax and cleaved caspase-3 at 12 h after OxyHb exposure compared to that of SAH group (Figures 6A,B). Furthermore, OxyHb exposure increased the ratio of Bax/Bcl-2 and administration of ATX-NPs decreased the ratio of Bax/Bcl-2 compared with experimental SAH group (Figure 6C). In addition, the results showed that the effect against apoptosis of Fe_3_O_4_/ATX/Transferrin was significantly higher than Fe_3_O_4_/ATX (Figures 6A–C).

**FIGURE 7:**
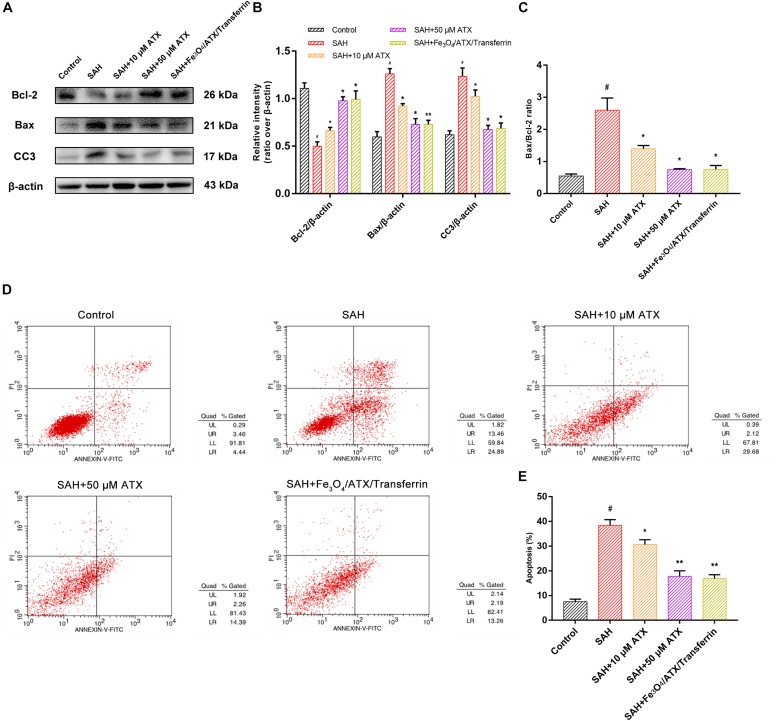
Comparison between ATX and Fe_3_O_4_/ATX/Transferrin treatment on neuronal damage in cultured neurons exposed to OxyHb. **(A)** Representative Western blots showed the expression of Bcl-2, Bax, and cleaved caspase-3 (CC3) at 12 h after OxyHb exposure in different groups. **(B,C)** Relative intensity analysis of Bcl-2, Bax, cleaved caspase-3 (CC3) and β-actin among all experimental groups, respectively. **(D)** Apoptosis detection results after OxyHb exposure were detected by flow cytometry. **(E)** The ratio of apoptotic cells in each group was measured. Values are expressed as the means ± SEM. ^#^*p* < 0.05 vs. Control group; ^∗^*p* < 0.05 vs. SAH group; ^∗∗^*p* < 0.01 vs. SAH group.

As displayed in Figures 6D,E, flow cytometry highlighted the presence of neuronal apoptosis at 12 h after OxyHb exposure. ATX-NPs, however, exhibited typical neuroprotective characteristics compared with SAH group and Fe_3_O_4_/ATX/Transferrin performed better consistent with the western blot analysis. These results confirmed that ATX-NPs protected against neuronal apoptosis after OxyHb exposure and transferrin significantly enhanced the effect through targeted delivery. More than that, Fe_3_O_4_/ATX/Transferrin revealed a superior neuroprotective effect compared with isodose free ATX and matched the efficacy from higher concentration group (50 μM ATX) (Figure 7).

### Cytotoxicity Study

The *in vitro* cytotoxicity of ATX-NPs was evaluated in neuron cultures at different concentration gradients. As a result, no significant differences of LDH activity were observed in these groups, suggesting that the fabricated nanoparticles had good biocompatibility, which demonstrated the viable application for ATX-NPs on neurons (Supplementary Figure S1).

## Discussion

Astaxanthin has been shown to display complex and multifaceted activities as an anti-oxidant, anti-inflammatory, and anti-apoptotic agent for treatment in EBI after SAH (Wu et al., 2014; Zhang et al., 2014a, b; Zhang et al., 2015). Despite its *in vitro* and *in vivo* efficacy, the therapeutic applications of ATX have been limited by its insolubility. Therefore, some studies have demonstrated higher efficacy and bioavailability of ATX on different paradigms in recent years (Anarjan and Ping Tan, 2013; Bharathiraja et al., 2017; Wang et al., 2017). To overcome this challenge, we have succeeded in employing Fe_3_O_4_ nanoparticles as drug delivery vehicles to C6 cells at the early stage (Tang et al., 2016).

For neurons, however, the efficiency of cellular uptake was greatly inferior to tumor cells after nanoparticle administration in preliminary experiments, which was probably due to the physicochemical property of nanoparticles, such as size, zeta potential, and shape. For this study, improved ATX-NPs with pore size of 10 nm and particle size of 20 nm were prepared. They exhibited excellent biocompatibility and endocytosis in the interaction with neurons, in corresponding to the study that the NPs with pore size of 2–50 nm were considered as the best candidate for delivery systems and theranostic applications (Bharti et al., 2015). Although some research pointed out that NPs with positive potential were easier to attach the cell surface, plenty of literature existed showing successful crossing of cell membrane with negative potential (Kou et al., 2018). Additionally, NPs with disk or rod or spherical shape indicated enhanced transcytosis and internalization via typical clathrin-mediated endocytosis (Herd et al., 2013).

Here, we showed that ATX could be well-loaded into Fe_3_O_4_ nanoparticles after physical adsorption. In the present study, PEG was selected to be the gatekeeping layer of ATX-NPs, resulting from the property which may induce immunogenicity and increase bioavailability of the polymer (Nie et al., 2009; Ding et al., 2014). In pretest, we have detected that the release of ATX from ATX-NPs could be significantly inhibited by PEG layer in PBS (not shown) but possibly increased under the intracellular environment of lysosomes (Hillaireau and Couvreur, 2009). Further, endosomal pathway including clathrin-dependent and -independent endocytosis is of vital importance for neuronal uptake with the absence of caveolae (Sahay et al., 2010). According to this manner, a stable release system can be achieved. In addition, excess autolysosomes activated in injured neurons after SAH may act as the demand stimulus to trigger the release of pharmaceutical ingredient to inhibit apoptotic progress (Chen et al., 2015, 2017).

The present study revealed that PEG polymer could be successfully prepared onto the ATX-NPs by solution blending and made the shape near-spherical with homogenous size distribution, observed in TEM photomicrographs. Meanwhile, the dispersity and stability of the fabricated NPs exerted a crucial part in biological applications of NPs as well (Henriksen-Lacey et al., 2016). In our DLS measurement, suspensions of PEG-coated ATX-NPs were steady during the period of 7 days with no observable alteration in the size distribution of NPs. The zeta-potential in water solution of ATX-NPs was about −40.8 mV, suggesting that PEG-coated NPs for drug delivery system were stable drug carries. Thereby, the obtained NPs strongly indicated that they may be a suitable substitute for former products with respect to neuronal uptake.

**FIGURE 8:**
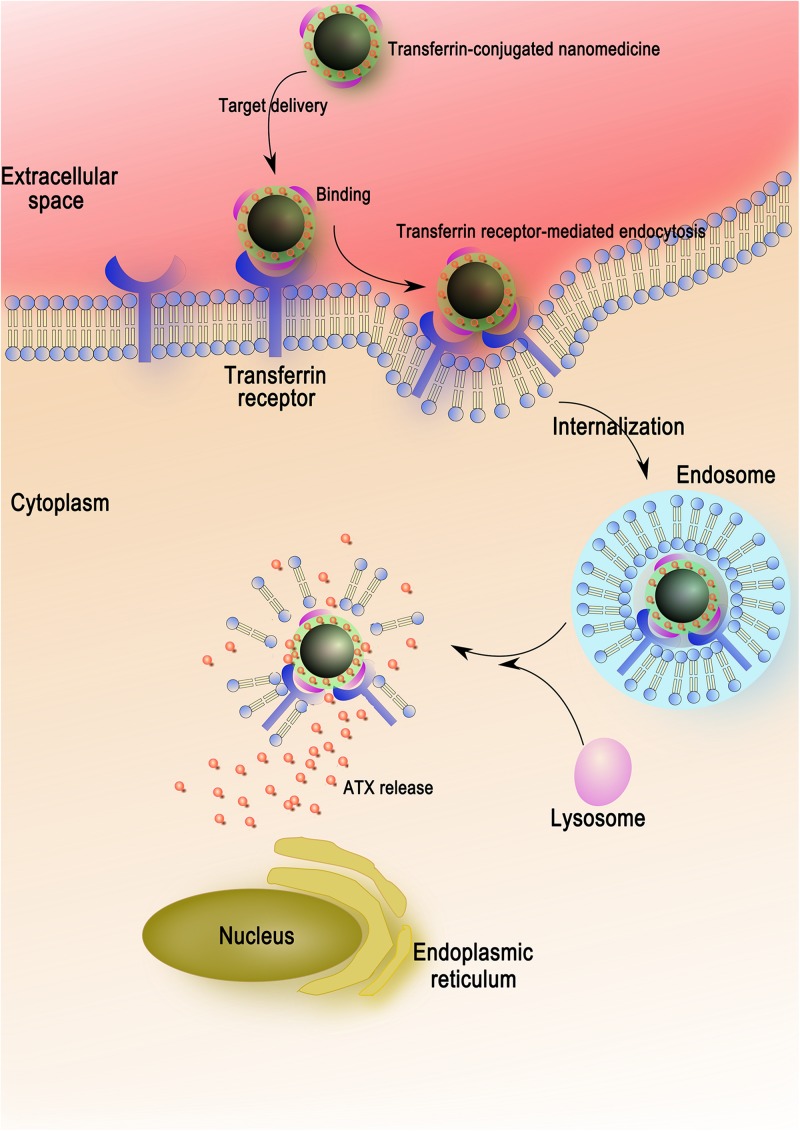
The entry mechanism of ATX-NPs in neurons via receptor-mediation. Firstly, the ATX-NPs in the medium are recognized by transferrin receptor of neurons. On the second stage, the nanoparticles are engulfed in membrane invaginations that are pinched off to form membrane-bound endosomes. Finally, the endosomes become mature and acidified fused with lysosomes, resulting in the degradation of nanoparticles and the release of astaxanthin.

For the sake of maximizing the ATX-NPs efficiency and minimizing the injury induced by experimental SAH, targeted delivery was thought to be a potential approach. It was well established that the transferrin receptor, which consisted of two linked 90 kDa subunits, was identified in choroid plexus epithelial cells, capillary endothelial cells, and neuron cells within brain cells (Moos and Morgan, 2000). It took part in mediating the delivery of iron to the brain in line with its highly expression in BBB (Jefferies et al., 1984; Moos and Morgan, 2000). As a transmembrane glycoprotein, moreover, transferrin receptor was the most widely studied receptor for the BBB targeting over the last decades (Li and Qian, 2002; Qian et al., 2002; Ulbrich et al., 2009; Jiang et al., 2012; Dixit et al., 2015). Thereinto, of major importance was the finding that transferrin receptor-targeting was superior to the other groups of targeting systems (IGFR, LDLR, and LRP) with regard to cerebral uptake of NPs (van Rooy et al., 2011). Notwithstanding, there were few reports concerning their application for target neurocytes through the BBB in CNS. For this reason, transferrin as a targeting ligand was evaluated for drug delivery in neuron cells. It consisted of two domains with alpha helices and beta sheets and owned high affinity to its receptor (Kaltashov et al., 2012). In conjugation with various nanomaterials, transferrin as efficient moiety for imaging and therapy of brain tumors has been extensively verified (Jiang et al., 2012; Dixit et al., 2015). In this work, successful conjugation of transferrin by EDC/NHS method markedly enhanced the transcytosis of ATX-NPs across the cortical neuronal membrane, making a therapeutic effect with sufficient concentrations (Figure 8). Both confocal and Biological microscopy confirmed the internalization, providing evidence that transferrin would be a promising tool to effectively facilitate the transport targeting neurons.

Synaptic loss and damage of neuronal populations in EBI after SAH occurs by stimulation of a cascade of pathophysiological events and multiple deleterious molecules rather than single pathogenic factor (Chen et al., 2014). Following SAH, cell apoptosis is widely provoked in smooth muscle, endothelial cell, neuron, and gliacyte. In particular, neuronal apoptosis might play an important role in SAH pathology (Sabri et al., 2008). Thereinto, the balance between Bax and Bcl-2 is fundamental for neuronal survival and death. In detail, overexpression of Bax usually causes mitochondrial membrane permeabilization, cytochrome c release, and caspase-3 and -9 activation, subsequently initiating apoptosis (Cahill et al., 2006). In primary culture of cortical neurons, OxyHb exposure induced significant neuronal apoptosis indicated by increased apoptotic marker Bax and cleaved caspase-3 and FCM positive signals (Figures 6, 7). Our results found that ATX-NPs at fixed doses of 6.7 μg/ml (corresponding to about 11 μM ATX) modulated these changes. They significantly provided protective effects on neurons at 12 h post-SAH characterized by decreased apoptotic markers and elevated cell survival. Also, higher protection observed in the presence of Fe_3_O_4_/ATX/Transferrin compared with free ATX strengthened the finding that ATX-NPs conjugated with transferrin could be effectively against SAH-induced neuronal death via blocking cell apoptosis.

It should be stressed that our study still has several limitations. At the outset, the confocal microscopy evaluation employed in this investigation was no sensitive enough to differentiate the uptake differences. Thus, more sensitive evaluation systems should be applied to allow the results more scientifically and accurately. Next, despite the neuroprotective effects of ATX-NPs and the enhanced efficiency by targeted delivery on neurons have been demonstrated, it was still unclear how the potential intramolecular interaction by ATX in cytoplasm led to alleviated neuronal apoptosis. In addition, primary neurons exposed to OxyHb were utilized as the *in vitro* model of experimental SAH, but SAH *in vivo* compassed complex pathophysiological mechanisms more than neurotoxic effect from initial bleed. Future studies exploring the impacts of ATX-NPs on SAH *in vivo* are required.

## Conclusion

Early brain injury, which is commonly known as the main reason of mortality after SAH, remains untreatable, mainly due to the permeable limitations of exogenous or endogenous molecules. In the wake of the fact that 99% of drugs cannot reach brain target regions, a nanotechnology-based method might offer a viable option for secure, efficient, and specific-target drug delivery. In the present study, we applied innovative targeted delivery system to administer ATX to primary cultured neurons. Our formulated nanoparticles exhibited favorable biostability and biocompatibility. As such, we herein for the first time compared two types of transportation toward neurons for the sake of potentials in ATX-NPs delivery. More importantly, ATX-NPs conjugated with transferrin successfully served as a comprehensive platform for targeted therapy, which was based on our prophase pharmaceutical studies. The incorporation of receptor-mediated targeting led to higher migration across membrane of ATX-NPs than their individual effects. Furthermore, compared with free ATX, the entrapped ATX was more stable and the effects obtained from ATX-NPs conjugated with transferrin occurred for a better capacity. Overall, the results demonstrated a significant improvement in neuronal survival after OxyHb exposure as well as the reduction in apoptotic markers, sufficiently exhibiting a new perspective for precision medicine in EBI after SAH. As *in vitro* experiments successfully applied, additional *in vivo* research needs to be validated and the underlying mechanisms concerning its exact role in CNS are required to be addressed in future.

## Data Availability

All datasets generated for this study are included in the manuscript and/or the Supplementary Files.

## Ethics Statement

All experimental protocols used for animals (including all surgical procedures) were approved by the Animal Care and Use Committee of Jinling Hospital and conformed to the Guide for the Care and Use of Laboratory Animals published by the National Institutes of Health.

## Author Contributions

QW, X-sZ, and X-mZ: conceptualization. L-lW and W-dX: formal analysis. XZ: funding acquisition, project administration, and writing—review and editing. X-mZ and BY: methodology. SC and X-lT: resources. Z-qY: writing—original draft.

## Conflict of Interest Statement

The authors declare that the research was conducted in the absence of any commercial or financial relationships that could be construed as a potential conflict of interest.

## Supplementary Material

FIGURE S1 The cytotoxicity study of ATX-NPs on neurons *in vitro*.

FIGURE S2 The cytotoxicity study of Fe_3_O_4_ *in vivo*.

TABLE S1 The original data of nanoparticle size.

TABLE S2 The original data of nanoparticle zeta-potential.
