# Supplementary figures and images for: Receptor-Mediated Delivery of Astaxanthin-Loaded Nanoparticles to Neurons: An Enhanced Potential for Subarachnoid Hemorrhage Treatment
Source: Front Neurosci. 2019 Sep 18;13:989. doi: 10.3389/fnins.2019.00989 (PMC6759683; doi:10.3389/fnins.2019.00989)

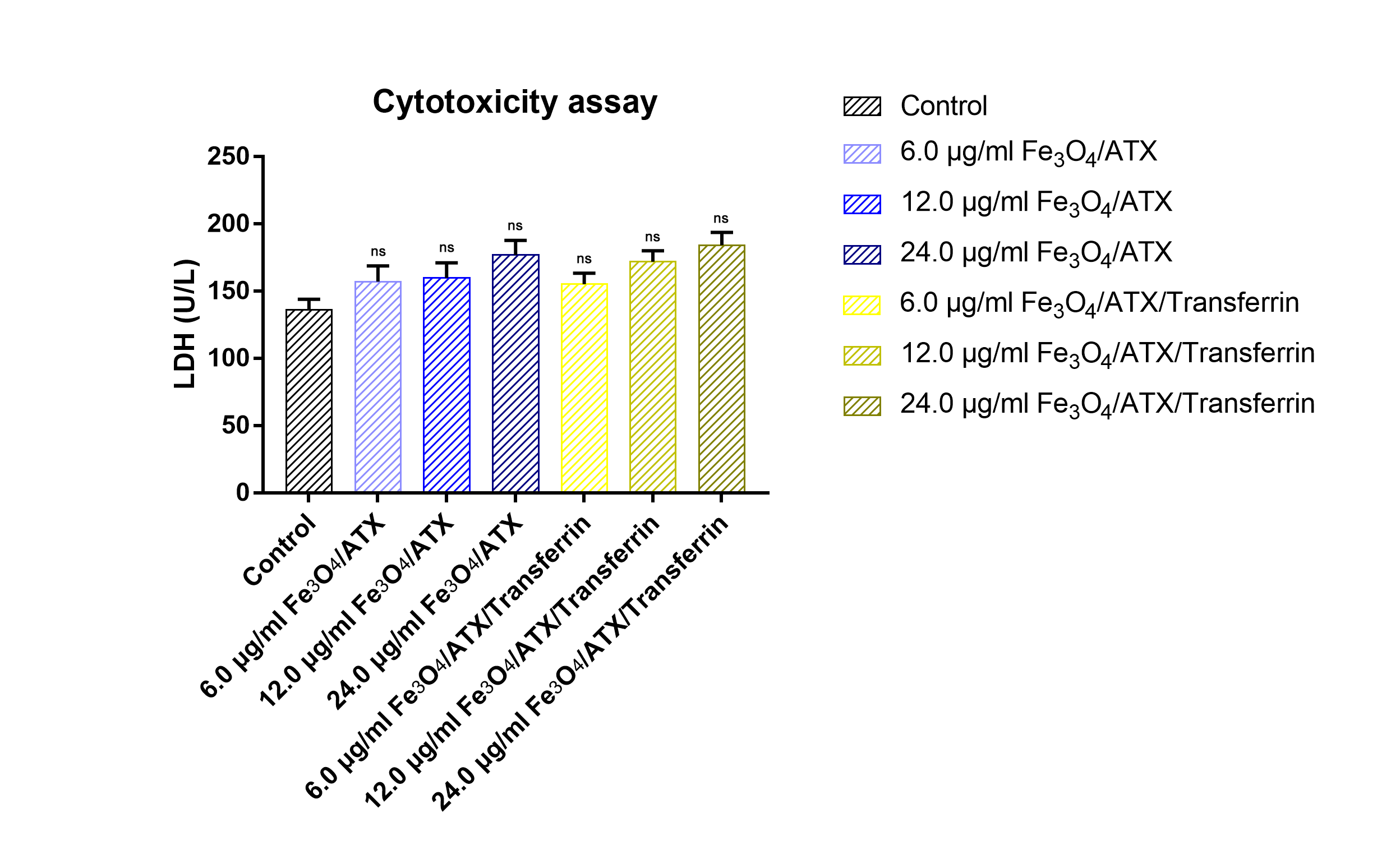

Supplement: FIGURE S1 — The cytotoxicity study of ATX-NPs on neurons in vitro. [file Image_1.TIF]

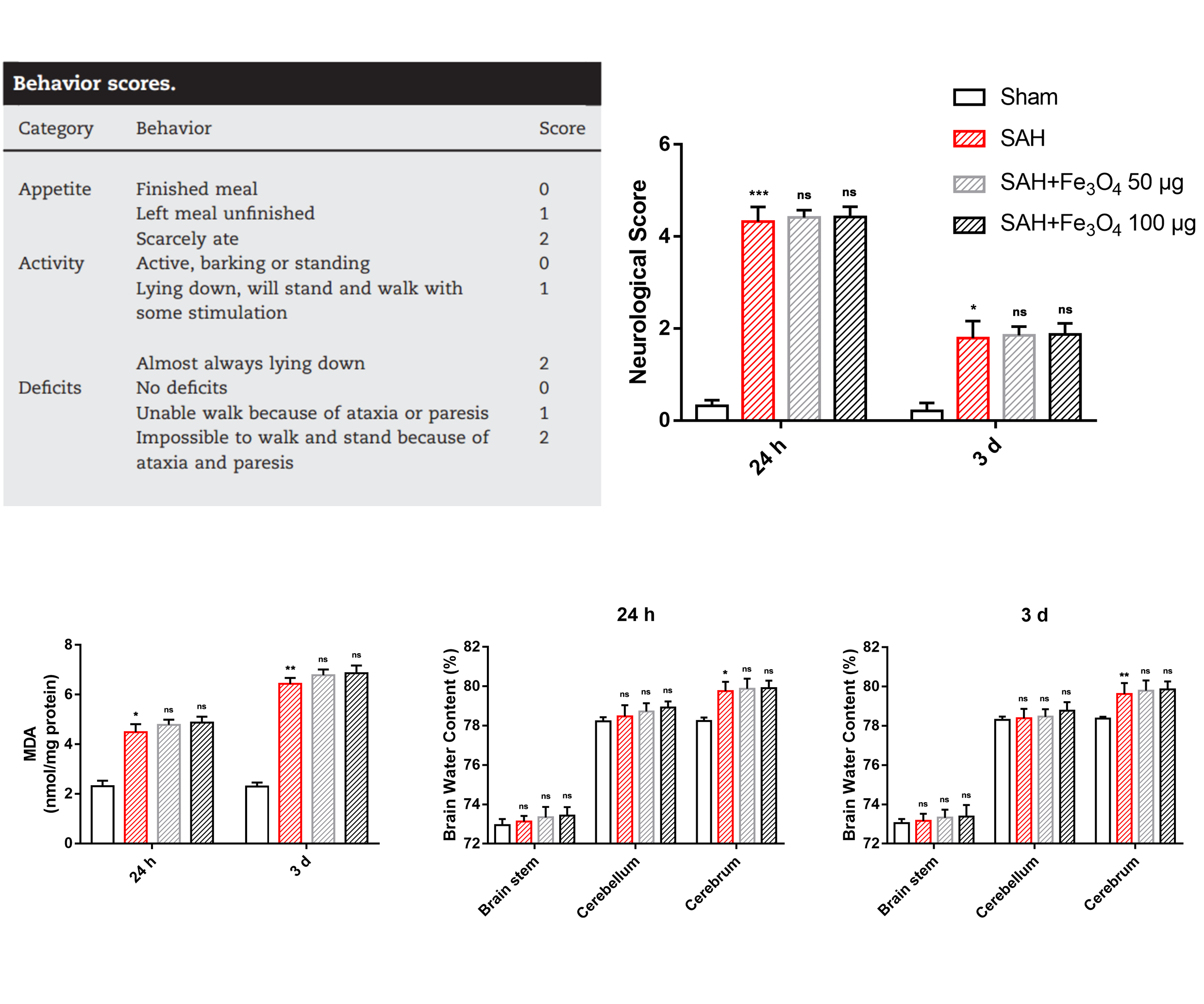

Supplement: FIGURE S2 — The cytotoxicity study of Fe3O4 in vivo. [file Image_2.TIF]
